# Bacterial Membrane Vesicles: Biogenesis, Functions, and Emerging Biotechnological Applications

**DOI:** 10.3390/microorganisms14030689

**Published:** 2026-03-18

**Authors:** Li Zhang, Yueyue He, Guilan Wang, Jiawei Sun, Yanwei Chen, Zhenling Wang

**Affiliations:** Cancer Center and State Key Laboratory of Biotherapy, Department of Biotherapy, West China Hospital, Sichuan University, Chengdu 610041, China; lizhang0103@126.com (L.Z.); heyy921@163.com (Y.H.); 13934863341@163.com (G.W.); sunjiawei20031003@163.com (J.S.); yanweichen1209@163.com (Y.C.)

**Keywords:** Bacterial membrane vesicles (BMVs), biogenesis, composition, function, applications

## Abstract

Bacterial membrane vesicles (BMVs) are non-replicative, bilayered nanostructures secreted by both Gram-negative and Gram-positive bacteria. Rather than being passive byproducts of cell envelope turnover, BMVs are increasingly recognized as regulated particles that selectively package proteins, lipids, nucleic acids, and other bioactive molecules. Through these cargos, BMVs mediate a wide range of biological processes, including bacterial stress adaption, intercellular communication, virulence delivery, and host immune modulation. In this review, we integrate recent advancements in understanding the molecular mechanisms underlying BMV biogenesis and composition and discuss how their heterogeneity contributes to their functional diversity. Beyond their biological roles, we critically examine the translational potential of BMVs in vaccine development, targeted drug delivery, cancer therapy, diagnostic tools, and biotechnological applications. However, significant challenges related to their safety, efficacy, and large-scale production must be addressed to realize their full clinical potential. We review recent progress and ongoing obstacles in the use of BMVs across various biomedical applications and propose strategies for their clinical translation.

## 1. Introduction

Bacteria release nanoscale, spherical structures known as bacterial membrane vesicles (BMVs) into their extracellular environment. Initially considered incidental byproducts of cell lysis or envelope turnover, BMVs are now recognized as an evolutionarily conserved and actively regulated secretion system found across nearly all prokaryotes, including both Gram-negative and Gram-positive bacteria [1]. In Gram-negative bacteria, these vesicles are often referred to as outer membrane vesicles (OMVs), which form from localized bulging of the outer membrane, encapsulating periplasmic components within a lipid bilayer. For many years, Gram-positive bacteria were thought to be incapable of vesicle production due to their thick peptidoglycan cell wall. However, accumulating evidence now demonstrates that Gram-positive species actively generate cytoplasmic membrane vesicles (CMVs) through coordinated cell wall remodeling and turgor-driven membrane extrusion processes [2,3]. Additionally, vesicle production has been documented in various archaeal species, suggesting that membrane vesiculation is a universal biological process that likely predates the evolutionary divergence of prokaryotic lineages [4]. More recently, the identification of outer-inner membrane vesicles (OIMVs) in Gram-negative bacteria, which encapsulate components from both the outer and inner membranes, has further expanded our understanding of BMV structural heterogeneity and underscored the mechanistic diversity of vesicle biogenesis pathways [5,6].

BMVs serve a wide array of functions that span microbial physiology, ecology, and pathogenesis. As specialized delivery vehicles, BMVs selectively package and transport proteins, lipids, nucleic acids, metabolites, and signaling molecules, thereby facilitating stress responses, horizontal gene transfer, quorum sensing, and biofilm formation [7,8,9,10,11]. In pathogenic bacteria, BMVs contribute to immune evasion, toxin delivery, modulation of host signaling pathways, and the spread of antimicrobial resistance determinants [12,13,14,15]. In contrast, BMVs from commensal and probiotic bacteria can exert beneficial effects on the host, promoting immune tolerance, reinforcing epithelial barrier integrity, and supporting mutualistic host–microbe interactions [16,17]. Beyond their endogenous biological roles, BMVs are increasingly viewed as modular and engineerable nanosystems with substantial translational potential. Their intrinsic stability, nanoscale dimensions, and ability to integrate immunostimulatory components with functional cargos make BMVs particularly promising for applications in vaccinology, antimicrobial therapy, targeted drug delivery, diagnostics, and cancer immunotherapy.

In this review, we provide a comprehensive and updated overview of BMV biology. We first summarize current models of BMV biogenesis, followed by an in-depth examination of vesicle composition. We then discuss BMV functions in bacterial homeostasis, stress adaptation, interspecies interactions, and host modulation. Finally, we highlight the emerging translational potential of BMVs in vaccines, drug delivery, diagnostics, and cancer immunotherapy, while critically addressing the key technical and regulatory challenges that must be overcome for clinical and industrial implementation. By integrating these perspectives, this review aims to provide an updated conceptual framework for BMV biology and illustrate how insights into bacterial vesiculation may inform future therapeutic and synthetic biology strategies.

## 2. Biogenesis of BMVs

**Figure 1:**
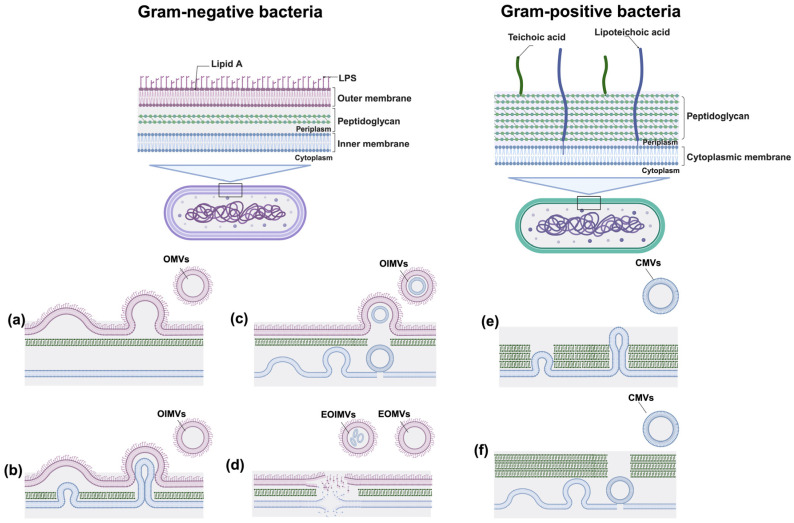
Biogenesis pathways of BMVs. In Gram-negative bacteria, BMVs are generated through outer and/or inner membrane blebbing and explosive cell lysis. (**a**) Outer membrane blebbing results in the formation of outer membrane vesicles (OMVs), typically driven by disturbances in cell envelope integrity, such as the insertion of hydrophobic molecules into the outer membrane or defects in peptidoglycan biosynthesis. Since the inner membrane remains intact during this process, OMVs generally lack cytoplasmic components. (**b**,**c**) Weakening of the peptidoglycan layer by endolysins can induce protrusion of the inner membrane into the periplasmic space, followed by pinching-off of the outer membrane, resulting in the formation of outer-inner membrane vesicles (OIMVs). (**d**) In contrast, explosive cell lysis is triggered by endolysin-mediated degradation of the peptidoglycan layer. Upon loss of cell wall integrity, bacterial cells round up and undergo lysis, and the resulting membrane fragments spontaneously self-assemble into explosive outer-inner membrane vesicles (EOIMVs) and explosive outer-membrane vesicles (EOMVs). Unlike OMVs and OIMVs formed by membrane blebbing, EOMVs and EOIMVs encapsulate cytoplasmic components in largely non-selective manner. (**e**,**f**) In Gram-positive bacteria, BMV biogenesis occurs through cytoplasmic membrane blebbing and/or a process termed bubbling cell death. Endolysin-mediated degradation of the thick peptidoglycan layer facilitates cytoplasmic membrane protrusion and vesicle release, leading to the formation of cytoplasmic membrane vesicles (CMVs). CMVs can encapsulate both membrane-associated and cytoplasmic components. Created with BioRender.com.

The biogenesis of BMVs is a tightly regulated and evolutionarily conserved process observed across diverse bacterial and archaeal lineages. Although the precise mechanisms of vesicle formation vary among taxa, all bacteria capable of vesiculation share the ability to remodel their cell envelope and to selectively package molecular cargo into nanoscale membranous structures (Figure 1).

### 2.1. BMV Biogenesis in Gram-Negative Bacteria

Virtually all Gram-negative bacteria constitutively release OMVs, which typically range from 20 to 400 nm in diameter [1]. OMVs are generated through localized outward blebbing and subsequent pinching-off of the outer membrane without compromising overall membrane integrity. Several non-mutually exclusive mechanistic models have been proposed to explain how outer membrane remodeling drives vesicle formation.

The earliest and most widely discussed model attributes OMV biogenesis to the weakening or loss of covalent linkages between the outer membrane and the underlying peptidoglycan layer [18,19]. Disruption of these cross-links, often mediated by alterations in lipoproteins such as Lpp, creates localized envelope instability. When the expansion of the outer membrane exceeds that of the peptidoglycan layer, membrane tension is relieved through outward protrusion, ultimately resulting in vesicle release.

A second model focuses on periplasmic crowding. The accumulation of peptidoglycan fragments, misfolded proteins, or aggregated periplasmic material increases localized osmotic or turgor pressure against the inner face of the outer membrane [15,19]. This pressure induces membrane curvature and bulging, leading to the release of OMVs enriched in periplasmic components. Environmental conditions that disrupt protein folding or peptidoglycan turnover, such as heat shock, oxidative stress, or antibiotic exposure, often trigger OMV formation via this pathway [11,20,21].

A third mechanism involves the localized enrichment of curvature-inducing molecules within the outer membrane. In *Pseudomonas aeruginosa* (*P. aeruginosa*), the quorum-sensing molecule *Pseudomonas* quinolone signal (PQS) intercalates into the outer leaflet of the outer membrane, sequestering divalent cations that stabilize negatively charged B-band lipopolysaccharide (LPS), thereby enhancing electrostatic repulsion and inducing outer membrane blebbing and OMV release [8,22]. While well characterized, this mechanism appears largely species-specific, as PQS production is predominantly restricted to *P. aeruginosa*.

More recently, disruption of outer membrane lipid asymmetry has emerged as a potentially conserved driver of OMV biogenesis. Impairment or downregulation of the VacJ/Yrb ATP-binding cassette (ABC) transporter system, particularly under iron-limiting conditions regulated by Fur, leads to the accumulation of phospholipids in the outer leaflet of the outer membrane [23]. This lipid enrichment increases lateral membrane pressure, promotes membrane curvature, and stimulates OMV formation. Lipid asymmetry-driven vesiculation has been demonstrated in *Haemophilus influenzae* (*H. influenzae*), *Vibrio cholerae* (*V. cholerae*), and *Escherichia coli* (*E. coli*), suggesting that regulated OMV release may trigger a general physiological response to nutrient limitation and envelope stress.

Beyond regulated outer membrane blebbing, Gram-negative bacteria can also generate BMVs through two alternative pathways: explosive cell lysis and inner membrane blebbing [6,24]. Explosive cell lysis is triggered under severe stress conditions, such as DNA damage, bacteriophage infection, antibiotic exposure, or host-derived lytic enzyme activity, which leads to peptidoglycan degradation and eventual rupture of the cell envelope [1]. Following lysis, membrane fragments self-assemble into vesicles that are either derived exclusively from the outer membrane, referred to as explosive OMVs (EOMVs), or from both outer and inner membranes, termed explosive outer-inner membrane vesicles (EOIMVs) [24]. Inner membrane blebbing, distinct from explosive lysis, represents a regulated form of vesiculation. For instance, we recently demonstrated that X-ray irradiation induces inner membrane blebbing in *P. aeruginosa*, followed by outer membrane pinching-off, leading to the formation of OIMVs [6]. A notable example of lysis-driven vesiculation occurs in *Shewanella vesiculosa* M7 (*S. vesiculosa* M7), where prophage-inducible explosive lysis generates complex, multilayered EOIMVs [25]. These structures often contain multiple internal vesicles and are morphologically distinct from OIMVs produced via membrane blebbing.

Collectively, these diverse biogenetic routes give rise to structurally and compositionally distinct vesicle subtypes with specialized biological functions. Canonical OMVs generated by outer membrane blebbing are typically enriched in LPS and periplasmic proteins, while largely lacking cytoplasmic material. In contrast, vesicles produced through explosive lysis or OIMV pathways encapsulate inner membrane lipids, cytoplasmic proteins, and nucleic acids, reflecting their origin in more extensive and disruptive envelope remodeling events.

### 2.2. BMV Biogenesis in Gram-Positive Bacteria

Despite lacking an outer membrane and possessing a thick peptidoglycan layer, Gram-positive bacteria are also capable of producing BMVs, commonly referred to as cytoplasmic membrane vesicles (CMVs) [1]. CMV biogenesis involves cytoplasmic membrane protrusion followed by passage through the rigid cell wall, a process enabled by localized wall remodeling or stress-induced weakening. This remodeling is largely mediated by autolysins and cell wall hydrolases, which transiently loosen peptidoglycan cross-links to facilitate vesicle extrusion. In *Staphylococcus aureus* (*S. aureus*), for example, the autolysin Sle1 has been shown to facilitate CMV release by locally weakening the cell wall structure [3]. Additional factors, such as teichoic acid composition, membrane fluidity, and lipid microdomain organization, further modulate vesiculation by influencing envelope rigidity and membrane curvature [26].

Studies in *Bacillus subtilis* (*B. subtilis*) has revealed that a prophage-encoded endolysin can induce CMV formation through a process known as “bubbling cell death” [2]. Unlike the complete explosive lysis observed in certain Gram-negative bacteria, *B. subtilis* cells undergoing this process often retain partial structural integrity, forming so-called “ghost cells” that contain intracellular vesicles. In *S. aureus*, amphipathic phenol-soluble modulins (PSMs) insert into the cytoplasmic membrane, perturb lipid bilayer organization, and promote curvature conducive to vesicle budding, while subsequent autolysin activity enables vesicle passage through the cell wall. Electron microscopy analyses consistently reveal continuous morphological connections between cytoplasmic membrane protrusions and released extracellular vesicles, supporting that CMVs are actively generated rather than being passive byproducts of cell death [3].

## 3. Composition of BMVs

**Figure 2:**
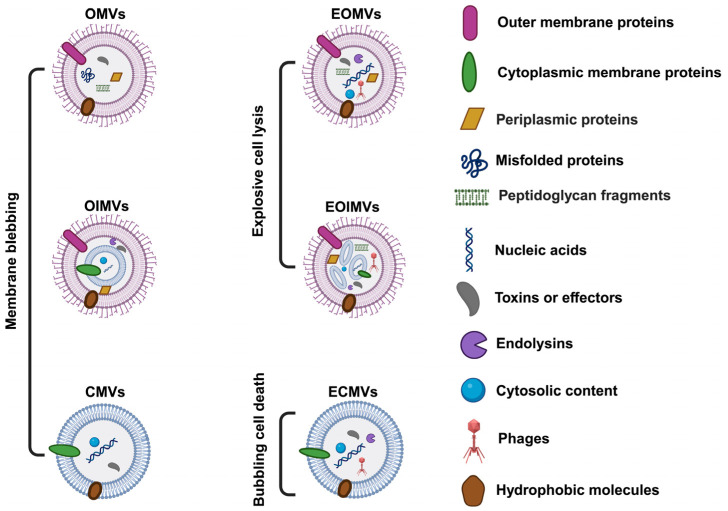
Types of BMVs with distinct structures and compositions. In Gram-negative bacteria, BMVs are generated via membrane blebbing or explosive cell lysis. OMVs are formed through blebbing of the outer membrane; their cargo lacks cytoplasmic components but is enriched in periplasmic and outer membrane proteins. OIMVs arise from inner membrane blebbing and, in addition to outer membrane and periplasmic proteins, contain inner membrane proteins, cytoplasmic proteins, and nucleic acids. EOMVs and EOIMVs are produced through explosive cell lysis, during which shattered membrane fragments self-assemble. Unlike OMVs, EOMVs and EOIMVs carry cytosolic contents, including genomic DNA. In Gram-positive bacteria, CMVs can be formed through membrane blebbing, where phenol-soluble modulins disrupt the cytoplasmic membrane, followed by CMV release through the cell wall after autolysin-mediated weakening of peptidoglycan cross-linking, and through bubbling cell death triggered by endolysin. CMVs contain cytoplasmic membrane proteins, cytosolic contents, and often toxins. ECMVs result from bubbling cell death induced by endolysins, stress-triggered autolysis, or exogenous peptidoglycan hydrolysis. ECMVs may carry endolysins, phages, or other lytic factors. Created with BioRender.com.

BMVs display a complex, heterogeneous, and highly dynamic molecular composition, shaped by their biogenesis pathways, the physiological state of the producing bacteria, and the surrounding environmental conditions (Figure 2). These nanoscale vesicles encapsulate a broad spectrum of biomolecules, including proteins, lipids, polysaccharides, and nucleic acids [27]. In Gram-negative bacteria, OMVs are typically enriched in outer membrane-derived components such as LPS, outer membrane proteins (OMPs), phospholipids, hydrophobic molecules, and virulence-associated factors [28]. In contrast, CMVs produced by Gram-positive bacteria predominantly contain cytoplasmic membrane proteins, cytosolic contents, membrane-associated enzymes, and secreted toxins [2,3]. Vesicle subtypes arising from alternative biogenetic routes, including OIMVs and CMVs, frequently encapsulate DNA, RNA, and small regulatory RNAs, contributing to bacterial communication, horizontal gene transfer, stress adaptation, and host immune modulation [1,6]. Accumulating proteomic and lipidomic evidence demonstrates that BMV cargo loading is highly selective rather than stochastic, highlighting their role as specialized and regulated vehicles for intra- and interspecies interactions [29,30,31,32,33]. Table 1 summarizes the distinct cargo profiles found in BMVs.

### 3.1. Proteins

Mass spectrometry-based proteomic analyses have revealed a remarkably diverse repertoire of proteins packaged within BMVs, offering critical insights into their biological functions and contributions to bacterial physiology and virulence [34]. BMV-associated proteins participate in essential processes such as environmental sensing, host cell adhesion, nutrient acquisition, and immune evasion [35,36,37,38]. Many BMVs contain abundant periplasmic and cytoplasmic enzymes, such as proteases, peptidases, nucleases, and β-lactamases, which enhance bacterial survival under stress conditions and facilitate adaptation to antimicrobial pressure [39,40,41,42]. Notably, the proteomic composition of BMVs varies substantially depending on bacterial species, growth phase, nutrient availability, and environmental cues. For example, during biofilm formation or nutrient limitation, *P. aeruginosa* OMVs are enriched in enzymes involved in extracellular matrix remodeling and stress tolerance, whereas *Salmonella enterica* serovar Typhimurium (*S*. *enterica* serovar Typhimurium) OMVs often contain immunomodulatory proteins that promote host colonization and immune manipulation [43,44,45].

Although canonical OMVs originate primarily from the outer membrane, increasing evidence supports the presence of inner membrane and cytoplasmic proteins within specific vesicle subpopulations. In *P. aeruginosa* and *S. vesiculosa*, prophage-encoded endolysins degrade the peptidoglycan layer, triggering explosive vesiculation and generating EOIMVs that encapsulate cytoplasmic proteins [24,25]. Similar phenomena have been observed in *Stenotrophomonas maltophilia* (*S. maltophilia*) and *E. coli* under genotoxic or antibiotic stress [46,47,48]. These alternative vesiculation pathways, which involve membrane rupture or dual-membrane extrusion, account for the presence of cytoplasmic proteins, metabolic proteins, and nucleic acid-binding factors, thereby markedly expanding the proteomic diversity and functional versatility of BMVs.

### 3.2. LPS

LPS is a major amphiphilic constituent of the outer membrane in Gram-negative bacteria and plays a central role in defining the biophysical and immunological properties of BMVs [22]. Composed of lipid A, a core oligosaccharide, and an O-antigen polysaccharide chain, LPS influence vesicle curvature, surface charge, and immunogenicity [49]. In classical OMVs, LPS is predominantly derived from the outer leaflet of the bacterial outer membrane and generally reflects the lipid A structure and O-antigen profile of the parental cell [50]. However, growing evidence indicates that LPS incorporated into OMVs can differ from that of the parental outer membrane in acylation state, phosphorylation pattern, and O-antigen chain length, pointing to selective enrichment during vesicle formation [51,52]. Such selective sorting may favor LPS chemotypes that promote membrane curvature, vesicle stability, or immune evasion. Notably, shorter or “rough” LPS variants are frequently associated with enhanced vesiculation and altered host immune recognition [53]. In contrast, OIMVs exhibit a hybrid lipid composition derived from both outer and inner membranes, incorporating LPS alongside phospholipids [54]. Although direct experimental evidence remains limited, this dual-membrane architecture, enriched in inner membrane proteins and cytoplasmic components, may enhance membrane fusion capacity or modulate interactions with host cells. Even greater LPS heterogeneity is observed in EOMVs, which arise from explosive cell lysis and therefore contain mixtures of intact and truncated LPS species [24]. Collectively, these structural variations shape vesicle morphology, immunogenic potential, and functional specialization.

### 3.3. Phospholipids

Phospholipids form the structural backbone of bacterial membranes and play a pivotal role in determining the physicochemical properties, curvature dynamics, and functional capacities of BMVs [55]. In Gram-negative bacteria, the outer membrane displays pronounced lipid asymmetry, with LPS dominating the outer leaflet and phospholipids, primarily phosphatidylethanolamine (PE), phosphatidylglycerol (PG), and cardiolipin, enriching the inner leaflet [56,57]. Although this asymmetry is partially retained during OMV formation, accumulating evidence indicates selective enrichment of specific phospholipid species within vesicles, highlighting the active and regulated nature of vesiculation [22]. Classical OMVs often exhibit increased levels of PE and PG, which facilitate membrane fluidity and curvature, while cardiolipin preferentially accumulates in regions of high curvature and promotes vesicle scission [40]. Environmental stressors, including nutrient limitation, antibiotic exposure, and osmotic imbalance, further remodel membrane lipid composition, generating OMVs with distinct phospholipid signatures [58,59,60]. In *P. aeruginosa* and *E. coli*, stress-induced increases in cardiolipin or unsaturated fatty acids correlate with enhanced vesicle production and improved envelope adaptation [61,62]. In contrast to classical OMVs, OIMVs incorporate substantial amounts of inner membrane material alongside outer membrane components, resulting in a dual-lipid architecture that modulates membrane fluidity, fusion potential, and the efficiency of effector molecule delivery [1]. EOMVs display the greatest lipid heterogeneity and are frequently enriched in inner membrane phospholipids and oxidized lipid species, reflecting their non-selective, fragmentation-driven origin [24].

Phospholipid diversity within BMVs carries important biological implications. Membrane charge, curvature stress, and fluidity collectively govern vesicle stability, interactions with host cells, and selective cargo packaging [63]. Moreover, specific phospholipids, including cardiolipin and lysophospholipids, can function as innate immune stimuli, directly shaping host inflammatory responses [64]. Overall, BMV phospholipid composition reflects a dynamic interplay among membrane architecture, environmental signals, and bacterial physiology.

### 3.4. Nucleic Acids

BMVs are increasingly recognized as active carriers of diverse nucleic acids, including chromosomal DNA, plasmid DNA, mRNA, tRNA, and small regulatory RNAs (sRNAs) [1,9,22,27]. Depending on vesicle subtype and biogenetic route, these nucleic acids may originate from cytoplasmic, periplasmic, or membrane-associated compartments [2,5,6,48]. Although early studies attributed vesicle-associated nucleic acids to contamination or cell lysis artifacts, it is now evident that many are selectively packaged and functionally relevant [6]. The nucleic acid cargo of BMVs plays crucial roles in bacterial communication, biofilm development, horizontal gene transfer, and host–pathogen interactions [9,24,65,66]. Bacteria-derived extracellular DNA, released during growth or biofilm formation, can associate with the surface of BMVs after vesicle release. In addition, DNA originating from cytoplasmic or periplasmic compartments may be incorporated into vesicles during biogenesis and thus protected within the vesicle lumen. In classical OMVs, extracellular DNA contributes to biofilm formation, antimicrobial tolerance, and immune activation [67,68]. OIMVs commonly harbor cytoplasmic or plasmid DNA, facilitating horizontal gene transfer and dissemination of antimicrobial resistance determinants [1,5]. Explosive OMVs and EOIMVs contain highly heterogeneous DNA fragments consistent with their lytic and non-selective origin [24,25]. Beyond DNA, BMVs also transport functionally active RNA species. sRNAs delivered by OMVs from pathogens such as *P. aeruginosa* and *Helicobacter pylori* (*H*. *pylori*) have been shown to modulate host immune responses, cellular metabolism, and inflammatory signaling pathways [69,70]. Vesicle-associated mRNAs may further support metabolic coordination within microbial communities or promote stress adaptation. Selective RNA loading is thought to involve RNA-binding proteins, lipid microdomains, and stress-responsive membrane remodeling, underscoring the intentional and regulated nature of nucleic acid incorporation into BMVs [6,71].

## 4. Functions of BMVs

**Figure 3:**
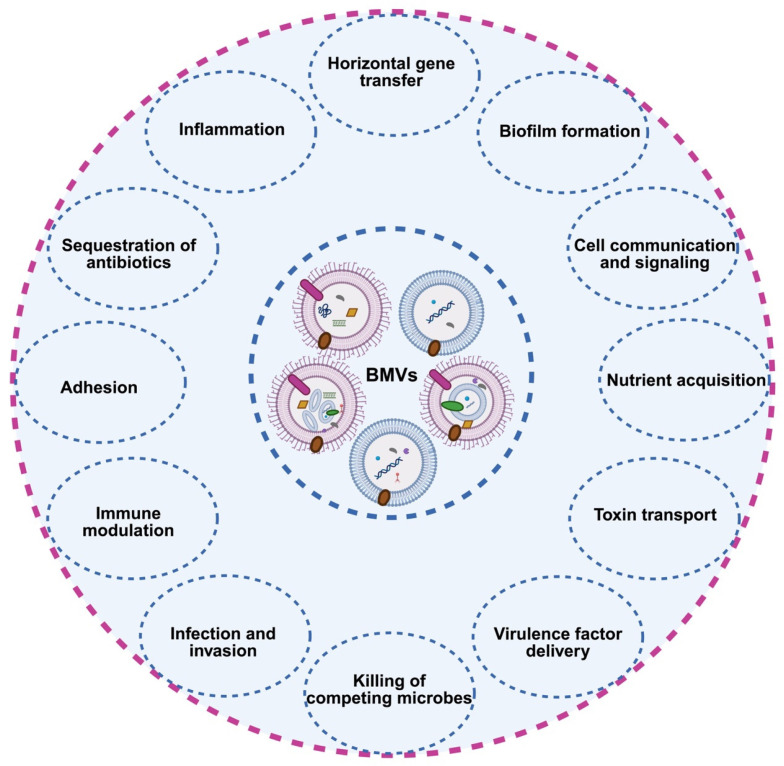
Biological functions of BMVs. BMVs play diverse roles in bacterial physiology and pathogenesis. These functions include horizontal gene transfer, biofilm formation and stabilization, intercellular communication and signaling, nutrient acquisition, toxin transport, and delivery of virulence factors. BMVs also mediate competitive interactions by inhibiting or killing competing microbes, facilitating host cell adhesion, infection, and invasion; modulating immune responses; and sequestering antibiotics. Created with BioRender.com.

BMVs are now recognized as multifunctional nanoscale structures that play central roles in bacterial physiology, intercellular communication, and host–pathogen interactions [27]. Once regarded as inert byproducts of cell growth or envelope turnover, BMVs are increasingly understood as actively produced, tightly regulated vehicles that selectively deliver biomolecules, sensing environmental cues, and modulate both microbial and host responses [72]. Their functional repertoire spans bacterial homeostasis and stress adaptation, virulence delivery, immunomodulation, microbiome dynamics, and host adaptation (Figure 3) [35,37,73].

### 4.1. Physiological Roles in Bacterial Homeostasis and Stress Adaptation

BMV production is closely linked to bacterial stress responses and the maintenance of cellular homeostasis. Beyond serving as a mechanism for eliminating toxic metabolites or misfolded proteins, BMVs selectively package specific cargos in response to environmental stressors. For example, under antibiotic pressure, ciprofloxacin-resistant *E. coli* produces OMVs enriched in proteins involved in energy metabolism and amino acid biosynthesis [74]. These vesicles can alter membrane potential and intracellular homeostasis in neighboring cells, thereby inhibiting or killing antibiotic-sensitive strains and conferring a competitive advantage to resistant populations.

BMVs also function as extracellular reservoirs of enzymes that mediate nutrient acquisition, redox regulation, and macromolecule degradation. Vesicle-associated hydrolases, such as chitinase and proteases, enable bacteria to exploit complex environmental substrates and support metabolic cooperation within microbial communities [22,27,35]. Exposure to antibiotics, oxidative stress, nutrient limitation, or membrane perturbation frequently triggers a marked increase in BMV release, supporting the view that vesiculation constitutes a rapid and adaptive response to envelope stress.

In addition, BMVs play a critical role in genetic exchange. By encapsulating chromosomal DNA, plasmids, and mobile genetic elements, BMVs facilitate horizontal gene transfer, thereby enhancing genetic diversity, population resilience, and adaptive potential [9,27,75]. Vesicle-mediated dissemination of genetic material has been documented in pathogens such as *P. aeruginosa* and *Acinetobacter baumannii* (*A. baumannii*), highlighting BMVs as important drivers of genomic plasticity and the spread of antimicrobial resistance [76,77,78]. Moreover, BMVs represent an integral component of the biofilm matrix. BMVs produced by *P. aeruginosa* are a significant and active source of extracellular DNA, which is crucial for biofilm stability, cell–cell cohesion, and the structural integrity of the community [42,72]. Studies have shown that biofilm-derived OMVs contain more DNA compared to their planktonic counterparts and can enhance DNA levels within the biofilm [79].

### 4.2. Pathogenic and Virulence-Associated Functions

In pathogenic bacteria, BMVs serve as efficient and highly targeted delivery vehicles for virulence factors, enabling manipulation of host cells without direct physical contact [36,37]. BMVs encapsulate diverse virulence determinants, including toxins, proteases, adhesins, and LPS, and gain access to host cells via endocytic uptake or membrane fusion. This vesicle-mediated delivery allows precise spatial and temporal modulation of host signaling pathways [22,27,50]. BMVs produced by pathogens such as *P. aeruginosa*, *H. pylori*, and *Porphyromonas gingivalis* (*P. gingivalis*) have been shown to disrupt epithelial barrier integrity, induce apoptosis, suppress innate immune responses, and create permissive niches for infection [50,80,81,82]. Importantly, BMVs can co-package multiple virulence factors, enabling synergistic effects upon host cell entry [83,84]. For instance, pore-forming toxins may facilitate cytosolic access of co-delivered proteases, thereby amplifying pathogenic outcomes [13]. In addition, BMVs can interfere directly with immune recognition and effector mechanisms by displaying host-mimicking surface structures or carrying enzymes that degrade immune components, such as complement factors, further enhancing immune evasion and pathogenic fitness [22,51,81].

### 4.3. Immunomodulatory Activities

BMVs are enriched in pathogen-associated molecular patterns (PAMPs) that are readily sensed by pattern-recognition receptors (PRRs) expressed on immune cells, leading to inflammasome activation and the production of proinflammatory cytokines [19,28,85]. For example, BMVs derived from *Listeria monocytogenes* (*L. monocytogenes*) strongly activate macrophages, inducing robust secretion of TNF-α, IL-6, and IL-1β [86]. Similarly, LPS-rich OMVs potentiate the stimulation of Toll-like receptors (TLRs), driving innate immune activation, cytokine release, and leukocyte recruitment [22,87].

Conversely, BMVs from certain bacteria exert immunoregulatory or immunosuppressive effects. BMVs released by *Enterococcus faecalis* (*E. faecalis*) MNC-168 activate the host STING pathway and promote type I interferon (IFN-β) production, suggesting potential applications in cancer immunotherapy [88]. More broadly, BMVs produced by commensal and probiotic bacteria contribute to the maintenance of immune homeostasis [89,90]. These effects include the induction of regulatory T cell (Treg) differentiation and the promotion of anti-inflammatory cytokines, such as IL-10 [91]. Recent studies have shown that polysaccharide A (PSA)-containing BMVs from *Bacteroides fragilis* (*B. fragilis*) induce a significant expansion of Treg populations, highlighting their potent immunoregulatory capacity. These OMVs also promote the production of anti-inflammatory cytokines, such as IL-10, which further contribute to the maintenance of immune homeostasis and mucosal immune tolerance [92]. Beyond proteins and lipids, BMVs also transport small RNAs and other regulatory molecules that modulate host transcriptional programs, further highlighting their context-dependent immunological roles at the host–microbiome interface [93].

### 4.4. Interbacterial and Interkingdom Communication

BMVs function as sophisticated molecular messengers that mediate communication both within bacterial communities and across biological kingdoms. They participate in quorum sensing by packaging and transporting signaling molecules such as PQS, and they distribute metabolites, enzymes, and genetic material to coordinate collective behaviors, including biofilm formation and virulence activation [27,67,72]. BMVs can also deliver antagonistic factors, such as bacteriocins, autolysins, and antimicrobial enzymes, to suppress competing species, as demonstrated in *A. baumannii* and *Myxococcus xanthus* (*M*. *xanthus*) [94,95].

Beyond bacterial interactions, BMVs exert broad effects on fungi, plant, and host cells. For instance, BMVs from pathogens such as *H. pylori* influence fungal morphology in co-culture models, and vesicles from marine bacteria can alter protist grazing behavior, highlighting the ecological breadth of BMV-mediated interkingdom signaling [75,96,97]. In plant-microbe interactions, BMVs are crucial for symbiotic outcomes. Rhizobia-derived BMVs can carry nodulation factors essential for initiating nitrogen-fixing symbiosis with legume roots [98]. In interactions with host cells, BMVs play multifaceted and pivotal roles in both health and disease. For example, BMVs from *Bacteroides thetaiotaomicron* (*B*. *thetaiotaomicron*) can exert beneficial effects by modulating host immune homeostasis and reinforcing intestinal barrier function [99]. Conversely, pathogenic BMVs are critical virulence vehicles that facilitate infection and disease progression. BMVs enable the targeted delivery of toxins and effector molecules directly into host cells. For example, *H. pylori* BMVs carry virulence factors such as adhesins (BabA, SabA) and the vacuolating cytotoxin VacA, which facilitate adhesion to gastric epithelial cells and subsequent induction of inflammation and apoptosis [100]. Together, these findings underscore the ecological, immunological, and therapeutic significance of BMVs in shaping microbial communities, host–microbe interactions, and disease outcomes.

## 5. Applications of BMVs

**Figure 4:**
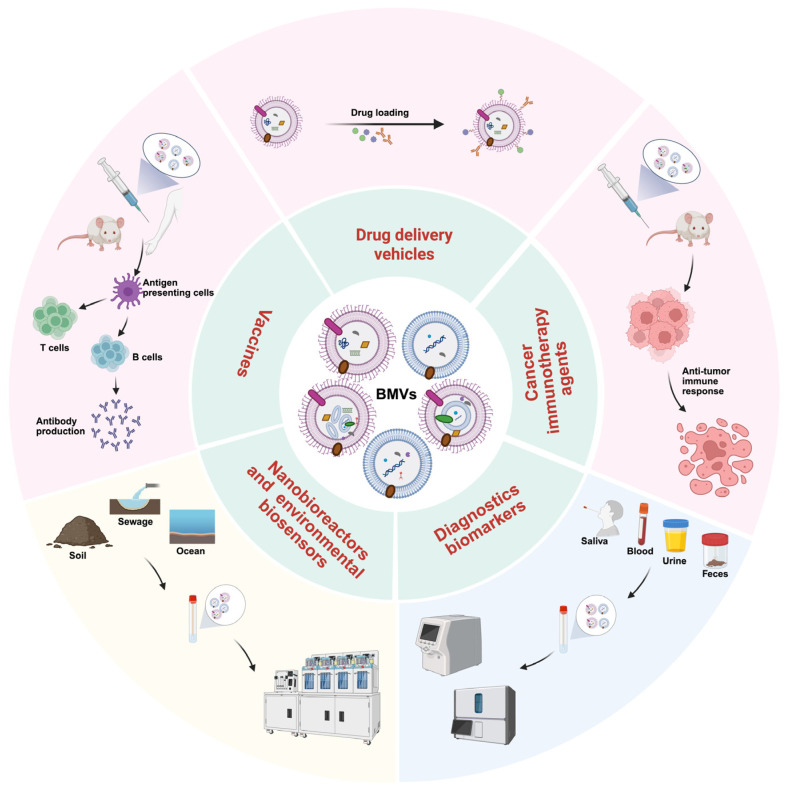
Biomedical applications of BMVs. BMVs hold significant potential in various biomedical applications, including as vaccine platforms, drug delivery vehicles, cancer immunotherapy agents, diagnostics biomarkers, nanobioreactors, and environmental biosensors. Created with BioRender.com.

As nanoscale lipid bilayer structures, BMVs, with their unique structure and biological properties, including intrinsic biocompatibility, structural stability, and the capacity to encapsulate diverse biomolecular cargos, have positioned themselves as versatile and highly adaptable platforms across biomedical and biotechnological fields. Importantly, BMVs naturally incorporate PAMPs, such as LPS, lipoproteins, proteins, and nucleic acids, enabling direct engagement with host immune systems and supporting applications in vaccinology, drug delivery, synthetic biology, diagnostics, and cancer immunotherapy (Figure 4) [101]. Table 2 summarizes clinical and preclinical studies of BMVs in vaccine and cancer immunotherapy applications from 2020 to 2026.

### 5.1. BMVs as Vaccine Platforms

BMVs possess intrinsic immunogenicity due to their enrichment in PAMPs, including LPS, lipoproteins, and peptidoglycan fragments. These components activate pattern recognition receptors (PRRs), such as TLR4 and TLR2, inducing robust innate immune responses that bridge to adaptive immunity [53]. The successful licensure of Bexsero^®^, a vaccine for protection against *Neisseria meningitidis* serogroup B (*N. meningitidis*) exemplifies this concept. This vaccine is based on OMVs bioengineered to display multiple protective antigens, including factor H-binding protein (fHbp), Neisserial heparin binding antigen (NHBA), and Neisseria adhesin A (NadA), demonstrating the capacity of BMVs to present a multivalent antigenic repertoire and induce broad protective immunity [102,103,104].

Advances in genetic and synthetic biology have greatly expanded both the safety and versatility of BMV-based vaccines [105]. A key innovation is the precise control of antigen localization, which critically shapes the immune response. Surface display of antigens using fusion scaffolds like ClyA, Lpp-OmpA, or autotransporter systems (e.g., Hbp and AIDA-I) preferentially promotes humoral immunity by enhancing B-cell accessibility [106,107,108,109]. In contrast, targeting antigens to the OMV lumen through signal peptides (e.g., PelB, OmpA, Lpp) favors intracellular antigen processing and MHC-I presentation, eliciting robust CD8+ T-cell responses that are essential for protection against intracellular pathogens [110].

Further engineering strategies aim to minimize reactogenicity while preserving immunogenicity. Producer strains such as *E. coli* BL21(DE3), which generates rough LPS lacking O-antigen, or ClearColi™, which synthesizes detoxified lipid A, are widely used to generate safer OMVs. Hypervesiculating strains created by disrupting envelope maintenance genes (e.g., Δ*mlaA*, Δ*nlpI*, Δ*tolR*) significantly increase vesicle yield, a critical parameter for scalable manufacturing. Furthermore, BMV-based vaccines can be administered via mucosal routes, such as intranasal or oral delivery, eliciting both systemic immunity and local secretory IgA responses—an advantage for vaccines targeting respiratory or enteric pathogens [111,112].

### 5.2. BMVs as Drug Delivery Vehicles

BMVs function as natural nanocarriers capable of stabilizing and delivering a wide range of therapeutic cargos, including antibiotics, chemotherapeutic agents, siRNA, and CRISPR–Cas components [113,114,115]. Their lipid bilayer structure protects encapsulated molecules from enzymatic degradation and enhances bioavailability. For instance, doxorubicin-loaded OMVs from *Klebsiella pneumoniae* (*K. pneumoniae*) preferentially accumulate in tumor tissue via the enhanced permeability and retention effect, resulting in targeted cytotoxicity in lung cancer models [116]. Similarly, *E. coli*-derived OMVs have been used to deliver siRNA for oncogene silencing or antimicrobial peptides to site of infection [117,118].

Through genetic and biochemical engineering, BMVs can be actively loaded with chemotherapeutics or genome-editing machinery, further broadening their utility in oncology and gene therapy [119,120]. BMV surfaces can be bioengineered via genetic modification or chemical conjugation to display targeting ligands or receptor-binding motifs, enhancing tissue specificity while reducing off-target toxicity [121,122]. Ligands such as affibodies, aptamers, or peptides can be conjugated to BMVs to enhance specificity for cancer or immune cells [123]. Notably, BMVs exhibit the capacity to traverse biological barriers, including the blood–brain barrier, enabling therapeutic delivery to the central nervous system [124,125]. Compared with synthetic nanocarriers such as liposomes or polymeric nanoparticles, BMVs offer superior biocompatibility and intrinsic biological functionality, supporting their use in precision and personalized medicine [73].

### 5.3. BMVs as Cancer Immunotherapy Agents

BMVs have emerged as powerful platforms for cancer immunotherapy due to their intrinsic immunostimulatory properties, nanoscale architecture, and high degree of engineerability [126,127]. A defining advantage of BMVs is their ability to potentiate innate immune pathways while simultaneously promoting adaptive antitumor immunity through efficient antigen delivery and cross-presentation [128,129]. PAMPs embedded in BMVs drive dendritic cell maturation and enhance antigen cross-presentation, ultimately inducing cytotoxic T-cell responses. Preclinical studies have shown that BMVs loaded with immunomodulatory cargos, such as STING agonists, effectively suppress tumor growth and metastasis in murine models [130]. Additionally, engineered OMVs displaying immune checkpoint inhibitors, such as PD-1 antagonists, can overcome therapeutic resistance by reprogramming the tumor microenvironment [131]. BMVs also synergize with established therapeutic modalities. For example, in photodynamic therapy, BMVs loaded with photosensitizers generate reactive oxygen species under light activation, inducing immunogenic cell death and amplifying antitumor immune responses [115]. Combination strategies integrating BMVs with chemotherapy or radiotherapy further enhance systemic immunity and abscopal effects by promoting tumor antigen release and immune priming [132].

Leveraging their natural tropism and immunogenicity, BMVs have also been successfully repurposed as in situ cancer immunotherapeutic agents. Upon systemic administration, OMVs derived from engineered bacteria preferentially accumulate in tumor tissues through the enhanced permeability and retention effect [133]. Within the tumor microenvironment, these BMVs are efficiently internalized by antigen-presenting cells (APCs), where their PAMP cargos trigger robust activation. This process promotes the production of key antitumor cytokines and chemokines, such as interferon-gamma (IFN-γ) and CXCL10, which are critical for recruiting and activating cytotoxic T lymphocytes and natural killer cells [121]. In murine models, this BMV-mediated remodeling of the tumor immune landscape has generated potent antitumor responses, capable of inhibiting tumor growth and, in some cases, achieving complete tumor regression [134]. Beyond serving as standalone immunostimulants, BMVs provide a versatile platform for engineered cancer vaccines. Through surface display systems, such as antigen fusion to CytolysinA (ClyA) or hemoglobin protease (Hbp), or luminal encapsulation strategies, OMVs can be functionalized to present tumor-specific or tumor-associated antigens, directing precise and potent adaptive immune responses against cancer cells [128,135]. Collectively, these advances position engineered BMVs as a multifaceted and highly promising modality in next-generation cancer immunotherapies, combining targeted delivery, potent immune activation, and extensive capacity for functionalization.

**Table 2:** Preclinical and clinical studies of BMVs in bacterial vaccines and cancer immunotherapy agents from 2020 to 2026.

Bacteria	BMV Type	Composition	Target	Status and/or Reference
Bacterial vaccines				
MenB	OMVs	rMenB and OMV NZ (Bexsero)	Gonococcal infection	Phase II (NCT04350138)
MenB	OMVs	rMenB and OMV NZ	Meningococcal infection	Phase IV (NCT06113198)
MenB	OMVs	OMV (DX-104)	Meningococcal infection	Phase I (NCT07395739)
MenB	OMVs	rMenB, OMV, multicomponent recombinant MenACWY	Meningococcal infection	Phase II (NCT01272180)
MenB	OMVs	MenABCWY (MenACWY lyophilized component, rMenB and OMV NZ)	Meningococcal infection	Phase II (NCT03587207)
MenB	OMVs	rMenB, OMV	Meningococcal infection	Phase III (NCT06446752)
Salmonella	OMVs	GMMA, mutant OMV	*S.* Typhimurium and *S.* Enteritidis infections	Phase II (NCT06213506)
Tumor immunotherapy agents				
*E. coli* W3110	OMVs	Mutant OMVs, aPD-L1 scFv and Luc	4T1, HEPA1-6	Preclinical [136]
*E. coli* BL21 (DE3)	OMVs	Mutant OMV, PD-1	MB49, EMT6	Preclinical [137]
*E. coli* BL21 (DE3)	OMVs	OMV, cL, AD	CT26, 4T1	Preclinical [138]
*E. coli* BL21 (DE3)	OMVs	Mutant OMVs, CpG, MSN-PEG/PEI	B16-F10, MC38, 4T1	Preclinical [139]
*E. coli* BL21 (DE3)	OMVs	OMV, ClyA-scFv	HCT-116, HT-29, 4T1, A549	Preclinical [140]
*E. coli* BL21	OMVs	OMV, TfR, LYTAC, PD-1/PD-L1	B16F10, CT26	Preclinical [141]
*E. coli* DH5α	OMVs	Mutant OMV expressing fibroblast growth factor	B16F10, TC-1	Preclinical [142]
*E. coli* W3110	OMVs	Mutant OMV expressing ectodomain of programmed death 1	B16 and CT26	Preclinical [131]
*E. coli* Rosetta (DE3)	OMVs	Mutant OMV plug-and-display tumor antigens	B16-F10, MC38, Pan 02, and B16-OVA	Preclinical [128]
*E. coli* T1	OMVs	OMV coated on NPs	EMT6, EMT-EGFP, and CT26	Preclinical [143]
*E. coli* DH5α	OMVs	Fusing tumor cell membrane and OMV, and coated on NPs	B16F10	Preclinical [144]
*S.* Typhimurium	OMVs	OMV, polymeric micelles	B16F10	Preclinical [145]
*Klebsiella pneumonia* ACCC 60095	OMVs	OMV, doxorubicin	A549	Preclinical [116]
*S.* Typhimurium, *S. aureus*	OMVs	Mutant OMV	4T1 and CT26	Preclinical [146]

### 5.4. BMVs as Diagnostics Biomarkers

BMVs represent valuable sources for diagnostic and biomarker discovery because they encapsulate and protect biomolecules that reflect the physiological state of their parental bacteria. BMVs isolated from clinical specimens, including blood, urine, sputum, and gastric fluid, carry pathogen-specific proteins, lipids, and nucleic acids that enable early infection detection, pathogen identification, and therapeutic monitoring [147,148]. For example, OMVs derived from *H. pylori* or *Mycobacterium tuberculosis* (*M. tuberculosis*) can be detected in serum using proteomic or nucleic acid-based assays, providing non-invasive biomarkers for disease diagnosing and progression assessment [149,150]. In parallel, engineered BMVs are being developed as biosensing platforms capable of signal amplification and molecular recognition [151].

### 5.5. BMVs in Biotechnology and Synthetic Engineering

Beyond therapeutic applications, BMVs serve as versatile tools in biotechnology and synthetic biology. Engineered BMVs can act as nanobioreactors by encapsulating enzymes, thereby enhancing catalytic efficiency, stability, and reaction specificity [151]. Hypervesiculating bacterial strains enable large-scale BMV production, supporting industrial biomanufacturing workflows [105]. In environmental biotechnology, functionalized BMVs are being developed as biosensors capable of detecting pollutants, toxins, or heavy metals [152]. For example, *E. coli* OMVs engineered to display metal-binding proteins have been applied to quality monitoring and environmental surveillance [153].

Despite these promising applications, several challenges remain before BMV-based technologies can be widely translated into clinical or industrial practice. One major limitation lies in the scalable and reproducible production of BMVs. Vesicle yield and composition are highly dependent on bacterial strain, culture conditions, and genetic modifications, which can introduce substantial batch-to-batch variability and complicate process standardization. In addition, BMVs are inherently heterogeneous in their composition and cargo content, carrying a complex and dynamic repertoire of proteins, lipids, and nucleic acids derived from the parental bacteria. Another important challenge involves the efficient and controllable loading of exogenous cargos, particularly large biomolecules such as nucleic acids, for which current strategies remain relatively limited. Furthermore, while BMVs possess natural tropism toward certain tissues or immune cells, achieving precise and predictable targeting in vivo remains an area requiring further engineering optimization [154,155]. Finally, issues related to biosafety, endotoxin-associated reactogenicity, and product standardization represent important considerations for clinical translation.

## 6. Safety and Quality Control in BMV Applications

The successful translation of BMVs into vaccines, therapeutics, and diagnostics requires rigorous control over safety, purity, and stability. As biologically derived nanoparticles that retain components of their parental bacteria, BMVs must meet regulatory standards comparable to those applied to advanced medicinal products. Key challenges include achieving high-purity isolation, attenuating the reactogenicity of immunostimulatory components such as LPS, and ensuring long-term stability during manufacturing, storage and distribution.

High-purity BMV production requires the effective removal of residual bacterial cells, membrane debris, genomic DNA, free cytosolic proteins, and surface appendages such as flagella and fimbriae. While ultracentrifugation remains widely used, it is limited by low throughput and the potential co-isolation of contaminants [156]. Ultrafiltration offers improved scalability but may retain high-molecular-weight aggregates [157]. High-resolution techniques, including density gradient centrifugation (DGC) and size-exclusion chromatography (SEC), yield superior purity [158]. For example, combining ultrafiltration with SEC increases purity by approximately 7-fold compared to ultracentrifugation alone, while DGC achieves the highest purity, with a 34-fold improvement [159]. Consequently, integrated multi-step purification workflows, typically combining ultrafiltration with chromatographic polishing, are increasingly adopted to meet regulatory requirements for clinical-grade BMVs [160].

A central safety concern is the intrinsic endotoxicity of LPS, particularly its lipid A moiety. Although LPS underpins the potent adjuvant properties of BMVs, excessive TLR4 activation can lead to pronounced reactogenicity or systemic toxicity [161]. Precise molecular engineering of lipid A biosynthesis offers a validated strategy to decouple endotoxicity from adjuvanticity. Genetic modification of late acyltransferase genes, such as *lpxL1* or *pagL*, enables the production of BMVs with attenuated endotoxicity while preserving immunogenicity. Specifically, *N. meningitidis lpxL1* mutant strains exhibit a roughly 100-fold reduction in endotoxin activity. However, *lpxL1* mutant LPS retains robust adjuvant activity, restoring immunogenicity of major outer membrane proteins to wild-type levels and eliciting bactericidal antibody titers 100-fold higher than those achieved with *lpxL2* mutant LPS [162]. Similarly, deletion of *msbB* (encoding a myristoyl acyltransferase) represents another promising clinical strategy. *Salmonella* and *E. coli* Δ*msbB* mutants produce penta-acylated lipid A with markedly reduced TLR4 activation and inflammatory potential yet preserve strong immunostimulatory capacity for vaccine applications [163,164]. Alternative strategies include the use of rough LPS mutants or engineered strains, such as *E. coli* BL21(DE3) Δ60, which generate simplified LPS structures and reduced proteome complexity, thereby improving safety profiles [165].

Long-term stability remains an essential consideration for BMV-based products. BMVs are susceptible to structural degradation, aggregation, and cargo loss under suboptimal storage conditions [166]. While frozen (−80 °C) or refrigerated (2–8 °C) storage generally preserves BMV integrity, repeated freeze–thaw cycles or exposure to elevated temperatures can compromise membrane structure and antigenicity [53]. However, lyophilized vesicles supplemented with trehalose have been shown to remain stable for over 12 months at 4 °C, providing a promising approach to enhance the long-term stability of BMVs [167]. Addressing these challenges will require systematic optimization of formulation buffers, cryoprotectants, lyophilization protocols, and cold-chain logistics to ensure consistent product quality throughout manufacturing and distribution.

## 7. Conclusions and Future Perspectives

BMVs have emerged as fundamental and versatile mediators of microbial physiology, ecological dynamics, and host–microbe communication. Once regarded as passive byproducts of cell growth or envelope turnover, BMVs are now recognized as actively regulated, evolutionarily conserved nanostructures produced by both Gram-negative and Gram-positive bacteria. Accumulating evidence demonstrates that vesiculation is not a uniform or incidental process but rather a tightly controlled phenomenon driven by multiple biogenetic routes, including localized membrane blebbing, perturbation of lipid asymmetry, autolysin- or phage-mediated cell wall remodeling, and stress-induced explosive cell lysis. Each pathway generates vesicle subpopulations with distinct molecular architectures, cargo profiles, and biological functions, collectively underscoring the remarkable structural and functional heterogeneity of BMVs.

Advances in high-resolution imaging, multi-omics profiling, and genetic perturbation strategies have profoundly reshaped our understanding of BMV composition and function. It is now evident that BMV cargo loading is highly selective rather than stochastic. Proteins, lipids, polysaccharides, and nucleic acids are differentially enriched according to vesiculation mechanisms, bacterial physiological states, and environmental pressures. This selective packaging underpins the diverse biological activities of BMVs, which extend from envelope remodeling and stress adaptation to interbacterial competition, biofilm stabilization, immune modulation, and targeted delivery of virulence determinants.

From a translational perspective, BMVs represent a highly promising yet still underexploited class of biologically derived nanomaterials. Naturally immunogenic OMVs have already achieved clinical success as licensed vaccines, and engineered BMVs offer a modular platform for antigen presentation, nucleic acid delivery, immune pathway modulation, and precision drug targeting. In biotechnology and synthetic biology, BMVs function as secretion vehicles, nanobioreactors, and biosensors, highlighting their adaptability across diverse application domains. However, realizing their full translational promise requires careful consideration of safety, endotoxin attenuation, large-scale purification, and long-term stability. Engineered BMVs are emerging as next-generation vaccine platforms and precision delivery vehicles, yet their clinical translation faces challenges including scalability, reproducibility, and biosafety. Standardization of isolation, characterization, and quantification methods is urgently needed to ensure consistency across studies and facilitate regulatory approval. In addition, minimizing the intrinsic toxicity of bacterial components, particularly LPS, remains a key step toward safe therapeutic application.

Looking forward, several emerging directions are poised to redefine the BMV field. The integration of BMV biology computational and synthetic engineering will enable the rational design of vesicles with tailored cargo composition, targeting specificity, and immunological profiles. This will be accelerated by cutting-edge computational and imaging technologies. For instance, AlphaFold3-guided design of autotransporter (T5SS) and other outer membrane protein scaffolds offers an exciting approach for optimizing surface display of antigens, improving the precision and efficacy of BMV-based vaccines. Additionally, single-vesicle cryo-electron tomography will play a crucial role in resolving vesicle heterogeneity and uncovering cargo-sorting mechanisms, which remain poorly understood. These imaging advances will provide deep insights into structure–function relationships and cargo-specific interactions, ultimately advancing the rational design of BMVs for therapeutic applications. Cryo-electron microscopy and single-vesicle analytical technologies will be instrumental in resolving vesicle heterogeneity, and uncovering cargo-sorting rules and structure–function relationships that remain poorly understood. As fundamental principles governing bacterial vesiculation continue to unfold, BMVs are positioned at the intersection of microbiology, immunology, nanotechnology, and translational medicine. Continued interdisciplinary efforts will not only deepen our understanding of microbial life and host–microbe interactions but also unlock innovative strategies for vaccinology, infectious disease control, cancer immunotherapy, and precision medicine.

## Figures and Tables

**Table 1:** The distinct cargo profiles found in BMVs.

BMV Type	Biogenesis Mechanism	Membrane Architecture	Major Molecular Cargo	References
OMVs	Localized outer membrane blebbing driven by envelope stress, lipid asymmetry, or periplasmic pressure	Single membrane derived from the outer membrane	LPS, outer membrane proteins, phospholipids, periplasmic proteins, toxins and effectors	[8,19,20,22,23]
OIMVs	Regulated inner membrane blebbing followed by outer membrane pinching-off	Dual-membrane vesicles containing both outer and inner membrane	LPS, phospholipids, outer membrane proteins, periplasmic proteins, cytoplasmic proteins, enzymes, DNA, RNA, toxins and effectors	[6]
EOIMVs	Prophage-mediated explosive cell lysis triggered by DNA damage or stress	Multilayered vesicles composed of fragmented outer and inner membrane	LPS, phospholipids, outer membrane proteins, periplasmic proteins, cytoplasmic proteins, enzymes, endolysins, DNA, RNA, phages, toxins and effectors	[24,25]
EOMVs	Membrane fragmentation during explosive cell lysis followed by self-assembly of outer membrane fragments	Single membrane derived from outer membrane fragments	LPS, phospholipids, outer membrane proteins, periplasmic proteins, cytoplasmic proteins, enzymes, endolysins, DNA, RNA, phages, toxins and effectors	[24,25]
CMVs	Cytoplasmic membrane protrusion through peptidoglycan following autolysin-mediated cell wall remodeling or prophage-mediated bubbling cell death	Single membrane derived from cytoplasmic membrane	Cytoplasmic proteins, enzymes, autolysins, DNA, RNA, PSMs, toxins and effectors	[2,3]

## Data Availability

No new data were created or analyzed in this study. Data sharing is not applicable to this article.

## Abbreviations

The following abbreviations are used in this manuscript:

BMVs	Bacterial membrane vesicles
OMVs	Outer membrane vesicles
CMVs	Cytoplasmic membrane vesicles
OIMVs	Outer-inner membrane vesicles
EOIMVs	Explosive outer-inner membrane vesicles
EOMVs	Explosive outer membrane vesicles
PQS	Pseudomonas quinolone signal
LPS	Lipopolysaccharide
DNA	DeoxyriboNucleic Acid
ABC	ATP-binding cassette
PSMs	Phenol-soluble modulins
OMPs	Outer membrane proteins
RNA	Ribonucleic Acid
PE	Phosphatidylethanolamine
PG	Phosphatidylglycerol
mRNA	Messenger RNA
tRNA	Transfer RNA
sRNAs	Small regulatory RNAs
PAMPs	Pathogen-associated molecular patterns
PRRs	Pattern-recognition receptors
TNF-α	Tumor necrosis factor-alpha
IL	Interleukin
TLRs	Toll-like receptors
STING	Stimulator of Interferon Genes
IFN-β	Interferon-beta
Treg	Regulatory T cell
IFN-γ	Interferon-gamma
CXCL10	CXC chemokine ligand 10
fHbp	Factor H-binding protein
NHBA	Neisserial heparin binding antigen
NadA	Neisseria adhesin A
ClyA	Cytolysin A
Lpp-OmpA	Lipoprotein-OmpA fusion protein
AIDA-I	Adhesin involved in diffuse adherence I
PelB	Pectate lyase B
MHC-I	Major Histocompatibility Complex Class I
IgA	Immunoglobulin A
siRNA	Small interfering RNA
CRISPR–Cas	Clustered Regularly Interspaced Short Palindromic Repeats/CRISPR-associated proteins
PD-1	Programmed Cell Death Protein 1
DGC	Density gradient centrifugation
SEC	Size-exclusion chromatography
